# The Organization of Mitochondrial Supercomplexes is Modulated by Oxidative Stress In Vivo in Mouse Models of Mitochondrial Encephalopathy

**DOI:** 10.3390/ijms19061582

**Published:** 2018-05-26

**Authors:** Mir R. Anwar, Amy Saldana-Caboverde, Sofia Garcia, Francisca Diaz

**Affiliations:** Department of Neurology, University of Miami Miller School of Medicine, Miami, FL 33136, USA; mir.anwar84@gmail.com (M.R.A.); asaldana@fiu.edu (A.S.-C.); sofiapgarcia@gmail.com (S.G.)

**Keywords:** mitochondrial supercomplexes, oxidative phosphorylation, antioxidants, COX10, Rieske iron sulfur protein, complex III, complex IV, blue native gel electrophoresis

## Abstract

We examine the effect of oxidative stress on the stability of mitochondrial respiratory complexes and their association into supercomplexes (SCs) in the neuron-specific Rieske iron sulfur protein (RISP) and COX10 knockout (KO) mice. Previously we reported that these two models display different grades of oxidative stress in distinct brain regions. Using blue native gel electrophoresis, we observed a redistribution of the architecture of SCs in KO mice. Brain regions with moderate levels of oxidative stress (cingulate cortex of both COX10 and RISP KO and hippocampus of the RISP KO) showed a significant increase in the levels of high molecular weight (HMW) SCs. High levels of oxidative stress in the piriform cortex of the RISP KO negatively impacted the stability of CI, CIII and SCs. Treatment of the RISP KO with the mitochondrial targeted antioxidant mitoTEMPO preserved the stability of respiratory complexes and formation of SCs in the piriform cortex and increased the levels of glutathione peroxidase. These results suggest that mild to moderate levels of oxidative stress can modulate SCs into a more favorable architecture of HMW SCs to cope with rising levels of free radicals and cover the energetic needs.

## 1. Introduction

The mitochondrial electron transport chain (ETC), located in the inner mitochondrial membrane, is composed of four multimeric enzymes (Complexes I to IV or CI–CIV) and two mobile electron carriers (coenzyme Q and cytochrome c). Reducing equivalents from the Krebs cycle (NADH and FADH2) are transferred through the ETC to molecular oxygen with concomitant translocation of protons from the matrix into the inter membrane space (IMS) through CI, CIII and CIV. The protons in the IMS are translocated back into the matrix by ATP synthase (complex V or CV) coupling the ETC to ATP production. This process is known as oxidative phosphorylation (OXPHOS).

Only in the last decade has the regulation of OXPHOS function begun to be understood. Cellular metabolic state and various posttranslational modifications appear to affect OXPHOS function [1,2]. Initially, it was thought that the complexes of the electron transport chain where scattered in the mitochondrial inner membrane and coenzyme Q and cytochrome c were mobile, diffusing through the membrane carrying electrons from one complex to the next. Over the years the solid state model, the liquid-state model and the plasticity model have been proposed to explain the organization of the ETC in the inner mitochondrial membrane (reviewed in [3]). Studies using mild detergent extractions, blue native gel electrophoresis and sucrose gradients indicated that the mitochondrial complexes of the ETC interact with each other to form supramolecular organizations that were named supercomplexes (SCs). Supercomplex assemblies have been observed in a wide variety of organisms including bacteria, plants, fungi and mammals. Mammalian SCs are composed mainly of complexes CI, CIII and CIV in different stoichiometry: CI+CIII; CI+CIII_2_; CI_2_+CIII_2_; CI+CIII_2_+CIV_1–4_; CIII+CIV and CIII_2_+CIV_1–2_. The CI+CIII_2_+CIV_1_ array has been termed the “respirasome”. A higher complexity level has also been proposed where supercomplexes also interact to form megacomplexes or “respiratory strings” [4,5,6]. In addition to CI, CIII and CIV, some supercomplex arrangements have been shown to contain CII, cytochrome c and coenzyme Q [7]. What was initially believed to be an artifact quickly gained acceptance in the field and a decade ago the first studies investigating the functionality of mammalian SCs were performed by Enriquez’s group [7]. They convincingly demonstrated that isolated respirasomes indeed “respired” by transferring electrons from NADH to oxygen [7]. Since then, numerous studies have focused on understanding the function and the regulation of SCs in health and disease. Factors involved in the assembly and organization of SCs have been the subject of intense investigation. For example, lipids like cardiolipin seem to be essential for stability of SCs [8] and proteins like Rcf1 in yeast and HIG2A in mammals, COX7a2L and MCJ/DnaJC15 have been described as regulators of SC assembly [9,10,11,12,13,14,15].

The electron transport chain is one of the major producers of reactive oxygen species (ROS) in the cell, particularly at the level of CI and CIII [16]. It has been proposed that one of the functions of SCs is to allow for a more efficient electron transfer conferring kinetic advantage by substrate channeling. A more efficient electron transfer will avoid diffusion of intermediates that could prematurely react with oxygen to form free radicals such as superoxide [17,18]. SCs would also enhance the stability of respiratory complexes particularly of CI and would limit the production of reactive oxygen species (ROS) [19].

Recent studies using cryo electron microscopy have been able to create structural models of the respirasome from porcine and ovine heart to determine the precise organization of the mitochondrial respiratory complexes [20,21]. Single-particle cryo electron microscopy studies revealed that CIII and CIV bind at the same side of CI and the studies identified those subunits that are involved in the interaction of the respiratory complexes [22]. The high-resolution structure revealed that not only cardiolipin but also phosphatidylcholine and phosphatidylethanolamine are involved in protein-protein interactions. Moreover, the model also suggests an alternative pathway for electron transfer within the respirasome [22]. More recently, structural data from the highest-order assembly of the respiratory chain has been obtained by cryo-electron microscopy in human cells. This megacomplex is composed of CI_2_+III_2_+IV_2_ with the CIII dimer encircled by two copies of CI and CIV [23]. Regardless of the knowledge gained in the field, it has been difficult to make certain generalizations due to conflicting reports in relation to assembly factors, substrate channeling and the existence of dedicated pools of CoQ and Cyt c for SCs. Recent reviews summarize some these findings [24,25,26].

The role of derangement of mitochondrial complex interactions and their pathophysiological significance in disease conditions remains elusive. Alterations in the supramolecular architecture of respiratory complexes were reported in rat cortex during aging [27,28]; in an animal model of severe heart failure [29]; in fibroblast from Parkinson’s disease patients harboring Pink1 mutations and in neuronal cultures from Pink1 and DJ1 KO mice [30]. Whether alterations in the supramolecular organization of the OXPHOS complexes result in significant physiological consequences remains unknown. Recently, Greggio and colleagues reported that exercise increased ETC complexes and SCs content in skeletal muscle of humans when compared to sedentary controls underscoring the importance of SCs arrangements in conditions of increased energy demands [31].

Many neurodegenerative diseases, stroke and aging have been attributed to oxidative stress induced by impaired mitochondrial function and increased reactive oxygen or reactive nitrogen species (ROS and RNS, respectively). CI, CII and CIII are the most prominent sites of free radical production [32]. CI and CII produce superoxide within the mitochondrial matrix, whereas CIII releases radicals into either the matrix or the intermembrane space [33,34,35].

Although physiological levels of either ROS or RNS function as signaling molecules, excessive amounts, usually generated during pathological states, produce oxidative/nitrosative stress [36,37]. If the impaired OXPHOS is not regulated, further increases in reactive species can ensue, causing extensive oxidative damage (i.e., glutathionylation of iron sulfur clusters present in many of the OXPHOS complexes, carbonylation or nitrosylation of tyrosine residues) [38].

We have studied the OXPHOS complexes interdependence and supercomplex assemblies in fibroblast cell lines deficient in UQCRFS1 or COX10 derived from our mouse models of CIII or CIV deficiency, respectively [39,40]. UQCRFS1 encodes for the Rieske iron sulfur protein (RISP), one of the catalytic subunits of CIII. COX10 is a farnesyl transferase that participates in heme *a* biosynthesis and is required for the maturation and stability of Cox1, one of the catalytic subunits of CIV. We found in both the RISP and the COX10 KO fibroblasts an unexpected strong pleiotropic effect on CI levels [39,40]. Exposure of RISP and COX10 KO fibroblasts to hypoxia abrogated the pleiotropic effect on CI uncovering a ROS dependent mechanism responsible for CI instability [39,41].

To gain insight into the regulation of SCs in oxidative stress conditions in vivo, we analyzed the stability of CI and SCs in two mouse models of mitochondrial encephalopathy previously shown to have different levels of oxidative stress caused by either a CIII or a CIV deficiency in neurons [42]. The neuron-specific RISP KO contained high levels of 8-hydroxyguanosine, SOD2 and other oxidative stress markers in the piriform cortex and to a lesser extent in cingulate cortex and hippocampus when compared to the same regions in the neuron-specific COX10 KO [42]. Analysis of the different brain regions by blue native gel electrophoresis revealed rearrangements of the architecture of SCs in tissues with moderate levels of oxidative stress. A significant increase in the levels of high molecular weight (HMW) SCs was observed in cingulate cortex of both COX10 and RISP KO, and in hippocampus of the RISP KO. In piriform cortex of the RISP KO, tissue with high levels of oxidative stress, the stability of CI, CIII and SCs was compromised and an antioxidant rescued the stability of the respiratory complexes and SC formation. Finding ways to maintain optimal mitochondrial function by stabilizing OXPHOS complexes and regulating SCs can provide a novel approach to control the formation of reactive intermediates when cells are exposed to metabolic stress.

## 2. Results

### 2.1. The Organization of Supercomplexes Is Modulated by Oxidative Stress

To understand the regulation of SCs in vivo, we examined the levels of CI and SCs in mitochondria from different brain regions (hippocampus, cingulate and piriform cortex) of neuron-specific COX10 and RISP KO mice. These neuron-specific conditional KO mice were created using the Cre-loxP system where the ablation of the respective gene was driven by the CaMKIIα promoter [42]. We previously showed that the COX10 KO had lower levels of oxidative stress markers than the RISP KO. Immunohistochemistry of brain sections with 8-hydroxyguanosine antibody (marker of nucleic acid damage) showed strong staining in the RISP KO particularly in the piriform cortex whereas the COX10 KO showed a weak stain. Western blots to detect protein adducts of lipid peroxidation or nitrosylation using specific antibodies against 4-hydroxynonenal (4-HNE) and N-tyrosine respectively showed that the RISP KO had elevated levels of these adducts when compared to controls. The elevated levels of 4-HNE and N-tyrosine in the RISP KO was already evident from an early age (1 month) [42]. The COX10 KO showed increased levels of protein oxidation at older ages (4 months). Both RISP and COX10 KO showed increased levels of SOD2 when compared to controls although the levels of this antioxidant enzyme in the COX10 were modest when compared to the RISP KO [42]. When comparing brain regions, hippocampus displayed lower levels of oxidative markers than cingulate and piriform cortex [42].

**Figure 1:**
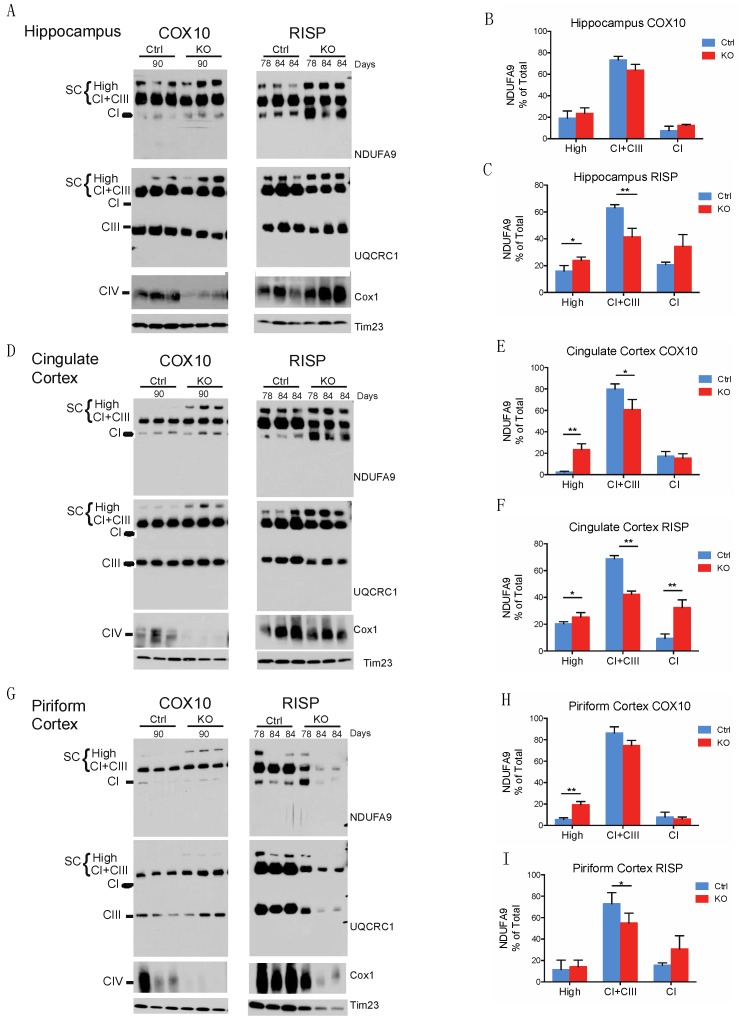
Blue native gel electrophoresis analysis of mitochondrial supercomplexes in brain regions of neuron-specific COX10 and RISP KO mice. Mitochondrial fraction was isolated from the different brain regions of control and KO mice at different ages (78–90 days). Mitochondrial proteins were extracted with digitonin and separated by blue native gel electrophoresis followed by western blot. CI, CIII and CIV were analyzed using NDUFA9, UQCRC1 and Cox1 antibodies respectively. Tim23 was used as mitochondrial loading control. Mitochondrial supercomplexes are indicated in the figure as SC (HMW and CI+CIII arrangements) in addition to free CI, free CIII and the monomer of CIV. Mitochondrial proteins were extracted from (**A**) hippocampus, (**D**) cingulate cortex and (**G**) piriform cortex from control and KO mice at ages indicated. (**B**,**C**,**E**,**F**,**H**,**I**) Graphs represent mean and standard deviation of levels of NDUFA9 normalized to Tim23 and expressed as percentage of total signal for hippocampus of COX10 (**B**) and RISP (**C**); cingulate cortex of COX10 (**E**) and RISP (**F**) and piriform cortex of COX10 (**H**) and RISP (**I**) mice. Antibody signals were quantified by densitometry of blots using ImageJ. (*) *p* < 0.05 and (**) *p* < 0.01 represents statistical significance, *n* = 3.

We obtained mitochondrial fraction using Percoll gradient from different brain regions from control, RISP and COX10 KO mice. Proteins were extracted with digitonin and separated by BN-PAGE followed by western blot to analyze SCs. Figure 1A shows SCs from hippocampus in both the RISP and the COX10 KO mice. There were no differences between the levels of CI, CIII and SCs in the COX10 KO when compared to control littermates assessed by NDUFA9 a CI subunit (Figure 1A,B) and UQCRC1 a CIII subunit (Figure 1A and Figure S1A) levels. NDUFA9 signal was distributed in high molecular weight (HMW) SCs, CI+CIII and free CI at the following relative levels: 19%, 73% and 7.5% of the total signal, respectively, for controls and 23.5%, 64% and 12.5% for the COX10 KO mice (Figure 1B). Similar results were obtained when blotting with antibodies against NDUFB8, NDUFV1, UQCRC2 and UQCRFS1, subunits of CI and CIII respectively (Figure S2A).

The hippocampus of the RISP KO showed a significant increase in the levels of HMW SCs, increase in free CI and a significant decrease in CI+CIII (Figure 1A,C). The percentage of NDUFA9 total signal distributed in SCs, CI+CIII and free CI was 16%, 63% and 21%, respectively, in controls and 23%, 41% and 34% in RISP KO. The decreased in CI+CIII observed in the RISP KO hippocampus was also reflected in the significant decrease of the levels of the CIII subunit UQCRC1 in this supercomplex arrangement (Figure S1B). This was also observed when using other subunit antibodies: NDUFB8, NDUFV1, and UQCRC2 antibodies (Figure S3A).

Analysis of cingulate cortex mitochondria revealed a significant increase in the levels of HMW SCs in both the COX10 and RISP KO mice (Figure 1D–F) as observed in the hippocampus of the RISP KO with NDUFA9 antibody. Similar to the hippocampus of RISP KO, the COX10 KO cingulate cortex has a percentage distribution of 2.6%, 80% and 17.4% for controls and 23.6%, 61% and 15.5% for COX10 KO (Figure 1D,E). The cingulate cortex of the RISP KO showed a redistribution of NDUFA9-containing SCs with a significant increase in the levels of HMW SCs and free CI and a significant reduction in the CI+CIII when compared to control mice. The distribution of the total signal for NDUFA9 into HMW SCs, CI+CIII and free CI was 21.7%, 69% and 9.4% respectively for controls and 25.3%, 42.25 and 32.4% for RISP KO cortex (Figure 1F). The levels of UQCRC1 were significantly increased in the HMW SCs. The HMW SCs increase was at the expense of a reduction of free CIII levels in the COX10 KO and a decrease in the CI+CIII in the RISP KO (Figure S1C,D). Similar results were observed when using antibodies against other subunits of CI and CIII (Figures S2B and S3B).

The piriform cortex of the COX10 KO showed similar results as the cingulate cortex with a significant increase in the HMW SCs with NDUFA9 antibody (Figure 1G,H). The percentage distribution of the NDUFA9 signal was 5.7%, 86% and 7.8% respectively for controls and 19.3%, 74.5% and 6.14% for COX10 KO. In contrast, the piriform cortex of the RISP KO showed a decrease in the levels of NDUFA9 and UQCRC1 in CI+CIII and a significant decrease in the levels of free CIII was observed (Figure 1G,I and Figure S1F). This was also reflected with antibodies against other CI and CIII subunits (Figure S3G).

The total levels of NDUFA9 and UQCRC1 signals normalized to Tim23 in all the COX10 KO brain regions were not significantly different than the ones in control mice, whereas in the RISP KO there was a significant increase in the levels of CI in cingulate cortex mitochondria and a significant decrease of CIII in piriform cortex but no differences in other regions (Figure S4). The increase in total levels of CI observed in cingulate cortex of the RISP KO was consistent with increased high molecular weight SCs and increased free CI (Figure 1D,F) showing a redistribution in the organization of the electron transport chain.

We also analyzed supercomplexes using two-dimensional (2D) BN-PAGE (Figures S2E,F,I and S3E,F,I) and results reflected what was observed in the first dimension. All the supercomplexes contained functionally active CI as indicated by in gel activity stain (Figures S2C,D,H and S3C,D,H).

The levels of CIV (monomer) were analyzed using the Cox1 antibody. As expected, the COX10 KO decreased CIV due to the instability of Cox1 in the absence of heme *a* (Figure 1A,D,G left panels). In the RISP KO mice the levels of CIV were not affected when normalized to Tim23 loading control (Figure 1G right panel). To confirm the efficiency of recombination in the RISP KO mice and determine residual levels of UQCRFS1, we also blotted membranes with specific antibody against this subunit (Figure S3A,B,G). KO mice had little RISP protein remaining and some signal was observed in overexposed blots as shown before [42].

Recent studies showed that CI in neurons is assembled preferentially into SCs whereas in astrocytes a higher proportion of CI is found free [43]. We isolated neurons from the whole adult brain of the COX10 and RISP KO mice at 3 months of age using the same MACS technology (Magnetic Activated Cell Sorting). In Figure S5, the isolated neurons show increased levels of NDUFA9 signal in HMW SCs, reduction in the levels of CI+CIII and increased levels of free CI in KO mice when compared to controls in both COX10 and RISP KO consistent with results shown in Figure 1. When neurons were compared to glial fraction (astrocytes, microglia and other cells) in control mice they contained higher levels of CI associated into SCs as described before [43]. The neuronal population obtained by MACS was highly pure and completely devoid of the astrocytic marker GFAP in western blot analysis. However, the glial fraction showed Tuj1 signal either from whole neuronal cells or axon terminals contamination (Figure S5).

### 2.2. MitoTEMPO Improved Stability of OXPHOS Complexes In Vitro and In Vivo

As mentioned above, our previous studies with the RISP and the COX10 KO fibroblast showed that the CI instability was associated with a ROS dependent mechanism [39,41] and use of some antioxidants (MnTBAP) but not others (n-acetyl cysteine) improved CI stability in the CIII deficient fibroblast [39]. Here we examine whether the mitochondria-targeted antioxidant mitoTEMPO (MT) was more effective in stabilizing CI in our RISP KO fibroblast than other conventionally and previously used ROS scavengers.

**Figure 2:**
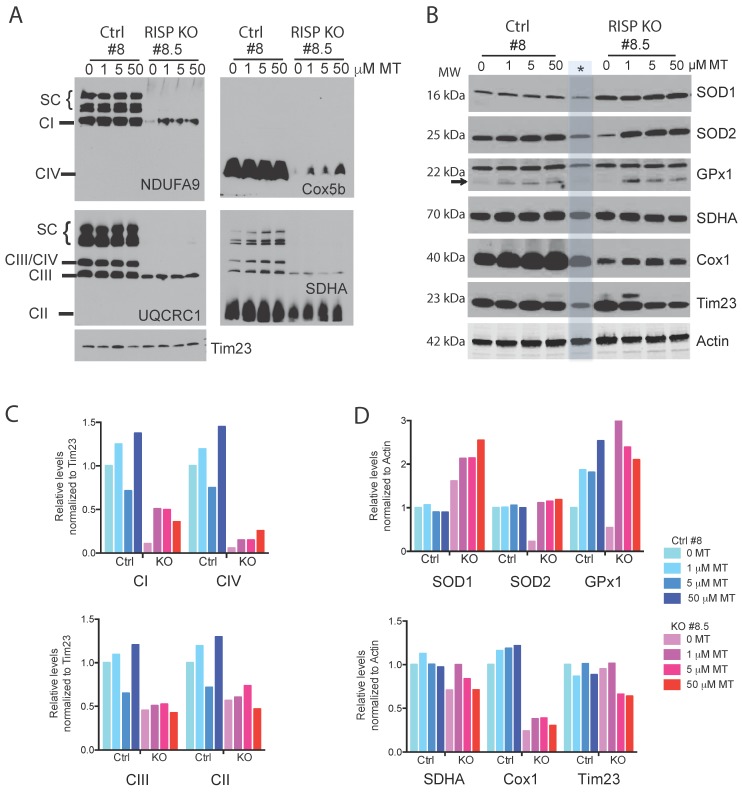
Mitochondrial supercomplexes in RISP fibroblasts treated with mitoTEMPO. Control (#8) and RISP KO (#8.5) mouse fibroblasts were incubated with different concentrations of mitoTEMPO (MT) for 24 h. Cell homogenates were prepared for the analysis of (**A**) SCs by BN-PAGE and western blot to detect SCs, CI, CIII, CIV and CII using antibodies against NDUFA9, UQCRC1, Cox5b and SDHA subunits respectively or (**B**) Steady-state level of various mitochondrial proteins by SDS-PAGE and western blot. Tim23 was used as loading control for BN-PAGE and actin was used as loading control for SDS-PAGE. Asterisk and dimmed blue bar denote unrelated sample to this study (Ctrl fibroblast exposed to hypoxia). (**C**,**D**) Densitometry of blots in (**A**,**B**).

We exposed control and RISP KO fibroblast to various concentrations of MT (1–50 μM) for 24 h and analyzed CI levels using BN-PAGE. Figure 2A,C show that MT improved the stability of CI in the RISP KO clone #8.5 (NDUFA9 panel). In addition to the CI defect, the RISP KO clone #8.5 also has a severe pleiotropic defect in CIV [39] and the mitochondrial antioxidant was able to increase the stability of CIV in the RISP deficient cells (Figure 2A Cox5b panel and 2C). RISP KO #8.5 fibroblasts treated with MT showed increases in the steady-state levels of cellular antioxidant enzymes SOD1, SOD2 and GPx1 but MT only increased GPx1 levels in the parental control cells #8. The levels of other mitochondrial proteins were increased at lower concentrations of MT (SDHA and Cox1) or were even decreased (Tim23) in the RISP KO #8.5 cells, whereas no effect was observed in the control cells with the exception of increased levels of Cox1 (Figure 2B,D). MitoTEMPO also stabilized CI in the COX10 KO #19 fibroblast (Figure S6 NDUFA9 panel) but did not improved stability of Cox1 and CIV in the absence of heme *a* (Figure S6 Cox1 panel).

**Figure 3:**
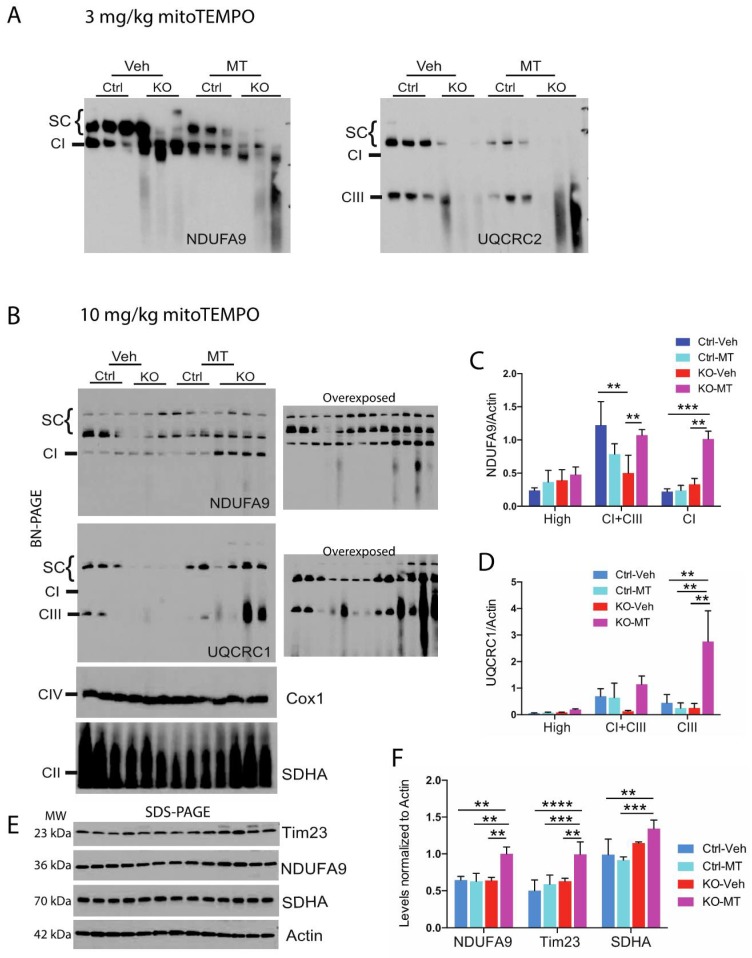
Analysis of mitochondrial supercomplexes in piriform cortex of the neuron-specific RISP KO mice treated with mitoTEMPO. Mitochondrial fraction from piriform cortex were obtained from control and RISP KO mice treated with either vehicle or with (**A**) low dose of 3 mg/kg/day or (**B**) high dose of 10 mg/kg/day of mitoTEMPO (MT). Mice were treated from weaning to 82–105 days of age. SCs were analyzed by BN-PAGE and western blot to detect SCs (HMW and CI+CIII), CI, CIII, CIV and CII using antibodies against NDUFA9, UQCRC1, Cox1 and SDHA subunits respectively. Low dose did not improved stability of respiratory complexes. Graphs represent the quantification of the levels of NDUFA9 (**C**) and UQCRC1 (**D**) normalized to actin in HMW SCs, CI+CIII, free CI or CIII respectively of the blots shown in (**B**). (**E**) Steady-state levels of some mitochondrial proteins in piriform cortex of vehicle and MT-treated mice by SDS-PAGE. Molecular weight (MW) for each protein is indicated in the figure. (**F**) quantification of blots in (**E**). Quantification of signal in blots was performed by densitometry analysis with ImageJ software. Protein levels were normalized to actin. Graphs represent the mean and standard deviation. Statistical analysis was performed by one-way ANOVA followed by Tukey’s multiple comparison test. (**) *p* < 0.01; (***) *p* < 0.001; (****) *p* < 0.0001 indicates statistical significance, *n* = 3–4.

Given the stabilizing effect of mitoTEMPO in CI and in the increase of cellular antioxidant enzymes in the fibroblast, we decided to treat the neuron-specific RISP KO mice with this mitochondria-targeted antioxidant and determine its effect on CI stability, supercomplex formation and in the oxidative stress of the piriform cortex. Mice were treated starting at weaning to 82–105 days of age with MT or vehicle at a dose of 3 mg/kg [44]. Unfortunately, at this dose we did not observe improvement in the stability of OXPHOS complexes in the piriform cortex mitochondria (Figure 3A). When we increased the dose to 10 mg/kg, a significant increase in the levels of NDUFA9 and UQCRC1 was observed in the KO treated with MT when compared to vehicle-treated mice. The levels of free CI and CI+CIII were significantly increased in NDUFA9 blots (Figure 3B NDUFA9 panel and 3C). Likewise, there was an increase in the levels of UQCRC1 in HMW SCs and CI+CIII and a significant increase in free CIII in RISP KO treated with MT (Figure 3B UQCRC1 panel and 3D). Interestingly, this improved effect on mitochondrial proteins was not observed in the control mice treated with MT (Figure 3F).

### 2.3. MitoTEMPO Improved Endogenous Cellular Antioxidant Defenses In Vivo

**Figure 4:**
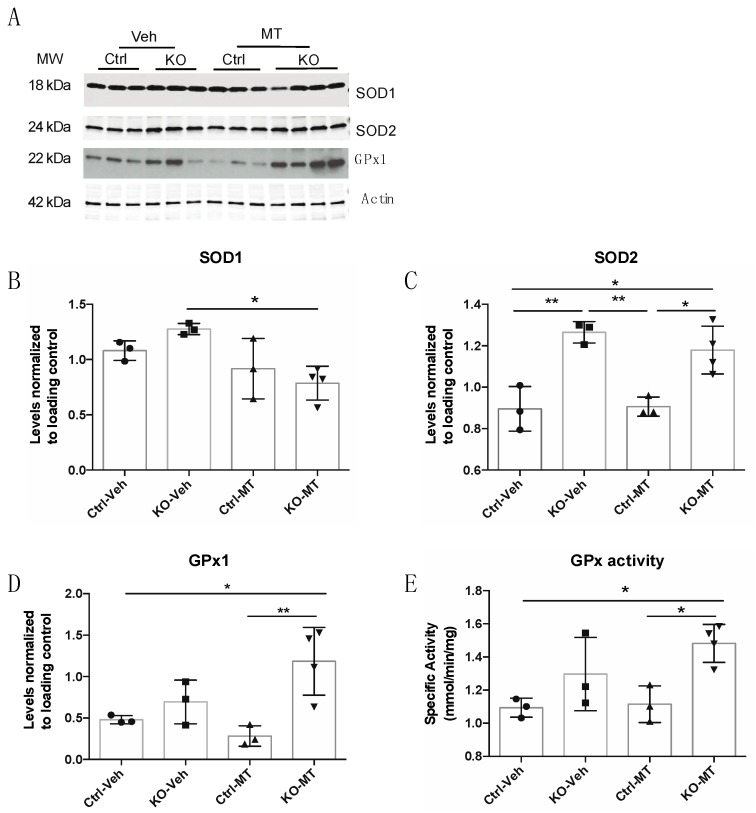
Analysis of cellular antioxidants in piriform cortex of the neuron-specific RISP KO mice treated with mitoTEMPO. (**A**) Steady-state level of antioxidant enzymes in control and RISP KO mice treated with vehicle or 10 mg/kg/day mitoTEMPO (MT) was determined by SDS-PAGE followed by western blot with specific antibodies. Molecular weight (MW) for each protein is indicated in the figure; (**B**–**D**) Quantification of levels of SOD1, SOD2 and GPx1 normalized to actin; (**E**) Enzymatic activity of glutathione peroxidase determined spectrophotometrically in piriform cortex homogenates. Each symbol represents one mouse (*n* = 3–4) and lines in graphs represent mean and standard deviation. Statistical significance was determined by one-way ANOVA followed by Tukey’s multiple comparison test. (*) *p* < 0.05 and (**) *p* < 0.01 indicate statistical significance.

To determine the effect of MT on the oxidative stress existing in the RISP KO, we examined the levels of various markers of oxidative stress in piriform cortex of treated mice. The steady-state levels of SOD1 were not altered between control and KO mice and MT decreased the levels of SOD1 in the KO mice when compared to the vehicle-treated mice (Figure 4A,B). The levels of SOD2 were significantly increased in both vehicle- and MT-treated KO mice when compared to control mice but there was no difference between KO vehicle and MT-treated KO mice (Figure 4A,C). There was an increase in the levels of glutathione peroxidase GPx1 (Figure 4A,D) in the RISP KO mice treated with MT. Increases in GPx1 levels were in agreement with the increase observed in glutathione peroxidase activity in the KO treated with MT when compared to vehicle-treated KO (Figure 4E).

## 3. Discussion

Various models have been proposed to explain the organization of the electron transport chain in the inner mitochondrial membrane (reviewed in [3]). More recently it was discovered that the respiratory complexes are not just dispersed in the membrane but they form associations into supramolecular structures named supercomplexes (SCs). Mitochondria are the major generators of reactive oxygen species (ROS) in the cell, in particular CI and CIII. It is believed that by organizing these respiratory complexes into supramolecular architectures, their close proximity will permit a more efficient substrate and electron transfer and will avoid the formation of reactive intermediates thus limiting the production of ROS [19]. The plasticity model proposed by Enriquez’s group predicts that SCs are dynamic structures that can change their composition in response to various stimuli or to metabolic changes adjusting to the cellular bioenergetic needs [45]. There are various factors that contribute to SCs assembly, including Rcf1/HIG2A, COX7a2L and MCJ/DnaJC15 [9,10,11,12,13,14,15]. In yeast mutants of Rcf1, the assembly of the respiratory chain into SCs is impaired leading to an increase in ROS production and maximal respiration was diminished indicating mitochondrial dysfunction during loss of supercomplex formation [9]. These results are consistent with the proposed role of SCs in preventing free radical formation and supporting more efficient respiration.

Our results showed a reorganization of the respiratory complexes and an increase in the levels of HMW SCs and free CI and a decrease in CI+CIII architectures in brain regions of the neuron-specific COX10 and RISP KO that displayed moderate oxidative stress. In tissue with high levels of oxidative stress, such as the piriform cortex of the RISP KO [42], we observed a dramatic reduction in the stability of CI, CIII and SCs formation.

The capacity of forming SCs depends first on the stability of its components. Mitochondrial respiratory complex interdependence has been observed in numerous cases. CI appears to be the most labile respiratory complex [46] and cultured cells with defects in CIII or CIV assembly have decreased CI [40,47,48,49]. Later we found that instability of CI in the CIII and CIV deficient cells was related to increased free radical production rather than the physical presence of fully assembled CIII and CIV [39,41].

How OXPHOS complexes are associated into supercomplexes is not completely understood. Ugalde’s group proposed that SCs are not formed by the association of fully formed complexes but that a CI subassembly intermediate first associates with CIII and CIV. Then CI assembly is completed by the incorporation of the NADH dehydrogenase module leading to the activation of the respirasome [50]. This hypothesis has been challenged by recent observations made by Nijtmans’ group who performed complexome profiling using bottom-up proteomics to analyze CI assembly [51]. The authors did not find CI assembly intermediates associating with CIII or CIV as suggested by Ugalde’s group. Authors indicate that their results were consistent with the previous proposal by Enriquez’s group that the respiratory complexes are fully assembled before associating into SCs [7]. Further studies are required to reconcile these contrasting findings.

The complete assembly process of CI remains elusive, however there is numerous evidence indicating that the process occurs in a modular rather than a sequential fashion [52]. CI can be divided into three functional modules: the N-module or catalytic (NADH oxidation), the Q-module (Ubiquinone or CoQ binding) and the P-module or proton pumping (membrane arm) [53]. There are 14 subunits in CI that represent the core of the complex and the rest of the 31 subunits have no definitive function [54,55]. The studies using gene-editing techniques to KO individually all the 31 accessory subunits of CI revealed that the loss of a particular subunit affects the stability of the other subunits in the same structural module and that 25 of the CI subunits are required for functional assembly [56].

To assess the levels of CI in our neuron-specific COX10 and RISP KO mice, we used antibodies against NDUFA9, NDUFB8 and NDUFV1 located in different modules of CI. NDUFA9 is an intermediate assembly subunit located between the peripheral arm and the membrane arm of CI. NDUFV1 is one of the central subunits of the NADH dehydrogenase catalytic module of the peripheral arm of CI. The catalytic module is added last on the proposed assembly pathway of CI although it is still unknown if the N-module is already fully or partially assembled when integrated to P/Q assembly intermediate [50,51]. Therefore, the presence of NDUFV1 in the blots of RISP and COX10 KO mice indicates a fully assembled complex either in free form or in SCs. NDUFB8 is one of the subunits located in the distal membrane domain (P module) and is incorporated into the complex around the same time than NDUFA9 is integrated [51,57]. NDUFB8 was recently identified as one of the subunits that mediates the oligomerization of CI, CIII and CIV in SCs [22].

The assembly of CIII also occurs in a modular fashion (reviewed in [58,59]). To assess the levels of CIII in our samples we used antibodies against UQCRC1, UQCRC2 and UQCRFS1 (RISP). During CIII assembly, the Rieske iron sulfur protein (RISP) and UQC11 are the last proteins to be incorporated into the complex [60,61]. In the RISP KO fibroblasts and brain tissue, a pre-CIII (already dimerized but lacking only two subunits: UQCRFS1 and UQC11) can be observed. This pre-CIII is incorporated into SCs as shown in the RISP KO blots ([39], Figure 1 and Figure S3).

In COX10 KO tissues, the total levels of CI and CIII were no different than the ones observed in control mice whereas in the RISP KO, we observed a significant increase in the levels of CI in cortex and a dramatic decrease in the stability of CIII in piriform cortex samples (Figure S4). This difference from the cultured models likely reflects the lower oxygen concentration in tissues (approximately 3–5%) when compared to cultured cells (approximately 20–21%) [62]. We also observed a difference in the distribution of CI (NDUFA9 signal) into CI+CIII and into high molecular weight SCs in KO mice from both models, in tissues with low to moderate levels of ROS. Interestingly, in those tissues with moderate levels of oxidative stress, there was a significant increase in the levels of HMW SCs. This was consistent with a significant increase of the UQCRC1 signal in HMW SCs, suggesting that redistribution into the respirasome might be an adaptive response to stabilize respiratory complexes and to prevent generation of reactive oxygen species [63]. Only when ROS levels rise is the formation of HMW SCs favored over the CI+CIII and its levels increase as observed in the cingulate cortex of the RISP KO.

The total levels of the free pre-CIII were not altered in hippocampus and cortex of RISP KO compared to control mice but were significantly affected by the high oxidative stress present in piriform cortex (Figure S1) [42]. Most of the pre-CIII is found associated with CI in the CI+CIII in the RISP KO cortex and piriform cortex. However, in cingulate cortex of the RISP KO with a milder oxidative damage than piriform cortex, we observed a significant increase of UQCRC1 signal in the HMW SCs (Figure S1D). 

The presence of HMW SCs has been described in other systems. Cybrid cells harboring a cytochrome b (cyt b) mutation accumulated HMW SCs at the expense of a decrease in CIII/CIV and free CIII [64]. The authors suggested that when the mutant CIII is assembled into SCs it results in less damage than when found alone. The cyt b mutation p.278Y > C enhanced superoxide production [64]. Preservation of HMW SCs has also been observed in Surf1 fibroblast derived from patients with a defect in this CIV assembly factor [65]. Cox1 signal was mainly found as an assembly intermediate or in SCs but little was found in the CIV monomer. In contrast, in Surf1^−/−^ mouse tissues there were very low levels of Cox1 in in HMW SCs (only detected in muscle and not in heart, brain and liver) and instead there is an accumulation of signal in the CIV monomer [65]. These differences in the distribution of Cox1 in SCs could be related to differences in species, to tissue bioenergetics or to differences in ROS production that could modify the distribution of OXPHOS complexes into the different supramolecular architectures. Unfortunately, the levels of oxidative stress were not characterized in the Surf1 deficient cell and tissues. In line with our findings, preservation of HMW SCs has also been observed in skeletal muscle of aged rats despite reduced amounts of low molecular weight SCs, CI, CIII and CIV when compared to young rats. This might constitute a compensatory mechanism of the aging muscle for a more efficient OXPHOS system, able to operate in a reduced level of metabolic intermediates and in the presence of a low level of individual complexes [28]. Similarly, studies in aged rat brain (30 months old) showed a small decline in the levels of CI+CIII+CIV_1–3_ of about 1.6-fold whereas a larger decline (2.8-fold) was observed in the levels of CI+CIII when compared to younger rats (5 months old) [27].

Differences in SC arrangements have also been reported in different mice strains. Buck et al. showed that the D2 mouse strain has abundant CI+CIII+CIV_2–3_ whereas these arrangements are barely detected in C57BL6 strains when analyzing brain tissue. C57BL6 is characterized by having stronger signals in CI+CIII+CIV and CI+CIII [66]. SC differences have also been documented in liver and heart tissues of various mice strains [67]. The difference found in mice strains have been also extended to the Cox7a2l which has been named SCAF1 for SC assembly factor 1. C57BL6 mice presumably have a mutation in this protein producing a short unstable version which impairs the formation of CIII+CIV SC [12]. Differences in SCs architecture extend to different species, tissues and cell lines. In our studies, we observed clear differences in the architecture of SCs between mouse brain and fibroblast cultures. In brain tissue, we mainly observed two bands: the HMW and the CI+CIII consistent with previous observations [66]. In contrast, the control RISP fibroblasts in addition to the CIII+CIV showed 3 additional bands in SC structures corresponding to CI+CIII, CI+CIII+CV and CI+CIII+CIV_n_ (in Figure 2A they can be seen clearly in the SDHA panel, blot that was previously blotted with UQCRC1). Recently, Lopez-Fabuel et al. found that in neurons, CI is assembled preferentially into SCs whereas astrocytes have higher levels of free CI [43]. The higher levels of free CI were associated with increased ROS production in astrocytes. To test if CI incorporation into SCs was associated with a reduction in the oxidative stress, the authors knocked-down NDUFS1 in neuronal cultures and observed a decrease in the association of CI with SCs that led to an increase in free radical production affecting mitochondrial respiration. When overexpressing NDUFS1 in astrocytes the authors observed an increase in the incorporation of CI into SCs and a decrease in ROS production [43]. Isolation of neuronal cell types from the COX10 and RISP KO mice showed similar results and confirmed that neurons of KO mice accumulated the more stable HMW SCs during oxidative stress when compared to controls (Figure S5).

To confirm that the dramatic decrease in OXPHOS complexes and SCs observed in piriform cortex of the RISP KO mice was due to the increased oxidative stress present in this brain region, we treated mice with the mitochondria-targeted antioxidant mitoTEMPO. The MitoTEMPO molecule contains the piperidine nitroxide TEMPO and the lipophilic triphenylphosphonium cation (TPP^+^), which allows it to accumulate several hundred-fold within mitochondria [68]. Consistent with the hypothesis that the distribution of SCs is regulated by the levels of free radicals in vivo, we observed a stabilization of OXPHOS complexes and SCs arrangements in the piriform cortex of mice treated with mitoTEMPO. Supporting the role of ROS in regulating SCs are the findings of Suthammarak et al. that reported than in *Caenorhabditis elegans* superoxide dismutase SOD2 interacts with SCs. They propose that SOD2 stabilizes CI+CIII+CIV structures and protects them from ROS damage [69]. The levels of SOD2 in the RISP KO mice were increased when compared to control mice as indicative of oxidative stress, however, mitoTEMPO did not have any further effect on SOD2 levels. Instead, mitoTEMPO increased the levels of glutathione peroxidase GPx1 and glutathione peroxidase activity in piriform cortex homogenates of RISP KO mice. SOD2 and GPx1 are found in the mitochondria. SOD2 converts superoxide radicals into hydrogen peroxide that are further catalyzed into water by GPx1, which uses glutathione to maintain redox balance in the cell.

In conclusion, our results suggest that reorganization of supercomplexes in vivo into a high molecular weight architecture in tissues with mild levels of ROS (COX10 KO cortex and piriform cortex) could constitute an adaptive response to stabilize CI into more favorable arrangement to cope with the rising levels of free radicals. Likewise, in tissues with moderate levels of ROS (RISP KO cortex) there is also an increase of the HMW SCs despite increased levels of free CI. In contrast, the stability of supercomplexes and respiratory complexes is dramatically affected in tissues with high levels of oxidative stress (RISP KO piriform cortex) and a mitochondria-targeted antioxidant treatment was able to stabilize OXPHOS complexes and SCs in vivo in a mouse model of mitochondrial encephalopathy.

## 4. Materials and Methods

### 4.1. Animal Husbandry

The mouse colony was maintained in a virus-free facility at the division of veterinary resources at the University of Miami under controlled room temperature and 12 h light/dark cycle. Mice were fed ad libitum with standard rodent diet. Experiments were performed according to protocols approved by the University of Miami Institutional Animal Care and Use Committee (#A-3224-01 effective 24 November 2015). Generation of RISP and COX10 neuron-specific conditional knockouts was performed using the Cre-loxP system. Deletion of genes was driven by the CaMKIIα promoter and described in [42]. COX10 KO mice was in a C57Bl6/J genetic background (more than 10 backcrosses) and RISP KO has to be maintained in mixed background (129svj and C57Bl6) because when backcrossed to C57Bl6 homozygous floxed UQCRFS1 mice did not produce any offspring. Mice were used independent of sex. We have not observed major sex differences in phenotype and biochemical analysis of these encephalopathy mouse models [42].

### 4.2. Treatment of RISP KO Mice with MitoTEMPO

Control and RISP KO mice were treated with intraperitoneal injections of mitoTEMPO [2(2,2,6,6,-tetramethylpiperidin-1-oxyl-4-ylamino)-2oxoethyl)triphenylphosphonium chloride]. Injections started at weaning until animals were euthanized at about 3 months of age. MitoTEMPO (MT) was obtained from either Enzo Life Sciences (Farmingdale, NY, USA; cat # ALX-430-160-M005) or Millipore Sigma (St. Louis, MO, USA; cat# SML0737-25MG). Stock solution of MT was prepared in DMSO at 20 mg/mL, aliquoted and stored at −20 °C. Before injection, drug aliquote was diluted in saline to obtain doses of either 3 mg/kg/day or with 5 mg/kg/2x day of MitoTEMPO for 5 days a week. For a 15 g mouse injected with the 5 mg/kg dose, 3.75 μL of MT stock was diluted in saline to final volume of 100 μL. Vehicle-treated mice were injected with DMSO in saline with the same amount of DMSO used in drug-treated mice.

### 4.3. Mitochondrial Fraction from Brain Tissues

Mitochondrial enriched fractions were prepared as described before [70] using frozen tissue from different brain regions (cingulate cortex, hippocampus and piriform cortex) of PBS perfused animals. Briefly, tissues were homogenized with a hand-held microcentrifuge tube homogenizer in 200 μL of 225 mM mannitol, 75 mM sucrose, 5 mM Hepes (pH 7.4), 1 mM EGTA, 0.2 mg/mL BSA containing complete protease inhibitor cocktail (Roche Diagnostics Corporation, Indianapolis, IN, USA), an aliquote was obtained for SDS-PAGE and western blot analysis and the rest was diluted to 1 mL placed in a 2 mL Dounce motor driven homogenizer and 30 strokes were used to completely disrupt the tissue. Homogenates were distributed into two Eppendorf tubes and filled to the top with the buffer mentioned above, mixed by gentle inversion and centrifuged at 500× *g* for 5 min. Supernatants were then centrifuged at 14,000× *g* for 10 min and the two pellets pooled by resuspending them in total volume of 200 μL of 12% Percoll™ in 225 mM mannitol, 75 mM sucrose, 5 mM Hepes pH 7.4, 1 mM EGTA. Sample was layered on top of 1 mL of 24% Percoll™, 225 mM mannitol, 75 mM sucrose, 5 mM Hepes (pH 7.4), 1 mM EGTA and centrifuged at 18,000× *g* for 15 min, discard top portion of supernatant (about 600 μL) and add more buffer (225 mM mannitol, 75 mM sucrose, 5 mM Hepes pH 7.4, 1 mM EGTA) to top of the tube, mix by inversion and centrifuge again at 18,000× *g* for 5 min. Supernatant was discarded and pellet resuspended in 1.5 M amino caproic, 75 mM Bis-Tris pH 7.0 buffer containing complete protease inhibitor cocktail (Roche) and then stored at −80 °C until used.

### 4.4. Western Blots

Samples were separated by SDS-PAGE using pre-casted 4–20% acrylamide gels (Bio-Rad, Hercules, CA, USA) and proteins transferred to nitrocellulose membranes. Membranes were blocked with 5% milk, incubated with primary antibody overnight followed with HRP-conjugated secondary antibody. Signal was developed using ECL and exposure to X-ray film. Antibodies used in this study were obtained from Abcam/Mitosciences (NDUFA9, NDUFB8, UQCRC1, UQCRC2, UQCRFS1, Cox1, Cox5b, SDHA and ATP5A); from Sigma (NDUFV1 and Actin); from Research Diagnostics Inc. (SOD1); from Antibody Verify (SOD2); from BD Bioscience (Tim23); and from Abgent (GPx1). All antibodies were used at a 1:1000 dilution in diluted blocking solution (0.25% *w*/*w* milk in PBST). Blots were quantified by densitometry were performed using ImageJ 1.49n software (National Institute of Health, Bethesda, MD, USA) and Tim23 or actin signals were used for normalization.

### 4.5. Blue Native Polyacrylamide Gel Electrophoresis (BN-PAGE)

Mitochondrial supercomplexes were extracted from the mitochondria enriched fraction described above using digitonin (Calbiochem) at a protein to detergent ratio of 1:8. Samples were incubated for 20 min on ice with digitonin and centrifuged at 21,000× *g* for 30 min. Supernatant were separated and 5% Serva G buffer was added to a 1:4 Serva G to detergent ratio. Samples were loaded in pre-casted 3–12% acrylamide native gels (Invitrogen, Grand Island, NY, USA) and separated as described before [71]. Proteins were transferred to PVDF membranes and blotted with 5% milk. Membranes were blotted with different antibodies and signal was developed using ECL and exposure to X-ray film. Quantification of western blots by densitometry was performed using ImageJ 1.49n software (National Institute of Health, Bethesda, MD, USA) and normalized to Tim23 to account for equal mitochondrial protein loading.

### 4.6. In Gel Activity Stain

To determine CI (NADH dehydrogenase) activity in gels, mitochondrial complexes were separated by BN-PAGE and gels incubated in 0.1 M Tris/HCl pH 7.4 containing 1 mg/mL nitro blue tetrazolium and 0.14 mM NADH at 37 °C [71].

### 4.7. Glutathione Peroxidase Activity

Glutathione peroxidase activity in piriform cortex homogenates was measured spectrophotometrically following the oxidation of NADPH at 340 nm using the Glutathione Peroxidase Assay kit (cat # 703102) from Cayman Chemicals (Ann Arbor, MI, USA).

### 4.8. Neuron and Glia Isolation

Neurons and glia were isolated from whole brain from control and KO mice at 3 months of age using MACS technology (magnetic activated cell sorting from Miltenyi Biotec, Bergisch Gladbach, Germany). Brain tissue was gently disrupted using the adult brain dissociation kit mouse (cat# 130-107-677) in C tubes in the gentleMACS Tissue Dissociator at 37 °C as indicated by the manufacturer. Neurons were isolated from obtained fraction by antibody-magnetic beads separation using the Neuron isolation kit mouse (Miltenyi Biotec cat# 130-115-389). Magnetic beads bind to non-neuronal cells and different cell types are separated using a magnet and MACS columns. Neurons are in the flow through fraction. Neurons and glia (fraction bound to magnetic column) were washed in PBS and cell pellets stored at −80 °C until used. Brains were processed individually. Final yield was about 2.6 × 10^6^ neurons per brain.

### 4.9. Cell Culture

The production of the RISP and COX10 knockout fibroblast cell lines derived from homozygous floxed mice was described in our earlier publications [39,40]. Cells were grown in high glucose Dulbecco’s modified Eagle’s media supplemented with 10% FBS, 1 mM pyruvate, fungizone, gentamycin and 50 μg/mL uridine at 37 °C in a 5% CO_2_ atmosphere. Cells were treated with 0, 1, 5 and 50 μM mitoTEMPO (Enzo, stock prepared in DMSO) for 24 h. The no drug (0 μM MT) treatment contained only DMSO at the same proportion as the 50 μM MT treatment. Cells were harvested and processed with 70 μL of 8 mg/mL digitonin/2.5 × 10^6^ cells to obtain membrane enriched fraction as described previously [71] and then stored at −80 °C until use.

### 4.10. Statistical Analysis

Results obtained were represented as mean ± standard deviation and two-tailed unpaired Student’s *t* test was performed to determine significance between two groups. When comparing more than two groups we used one-way ANOVA followed by Tukey’s multiple comparison test. *p* < 0.05 was considered statistically significant. Experiments performed with fibroblast in tissue culture were performed in 2 to 3 independent experiments. For mice samples, at least three biological replicates were analyzed.

## Supplementary Materials

Supplementary materials can be found at http://www.mdpi.com/1422-0067/19/6/1582/s1.
