# Supplementary material for: The Organization of Mitochondrial Supercomplexes is Modulated by Oxidative Stress In Vivo in Mouse Models of Mitochondrial Encephalopathy
Source: Int J Mol Sci. 2018 May 26;19(6):1582. doi: 10.3390/ijms19061582 (PMC6032222; doi:10.3390/ijms19061582)
Supplement: Supplementary file 1 [file ijms-19-01582-s001.pdf]

International Journal of *Molecular Sciences*

Article

The Organization of Mitochondrial Supercomplexes is Modulated by Oxidative Stress in vivo in Mouse Models of Mitochondrial Encephalopathy

Mir R. Anwar [†], Amy Saldana-Caboverde [†], Sofia Garcia and Francisca Diaz [*]

Department of Neurology, University of Miami Miller School of Medicine, Miami, Florida 33136, USA.

* Correspondence: fdiaz1@med.miami.edu

† Those authors contribute equally to this study.

Supplementary Materials

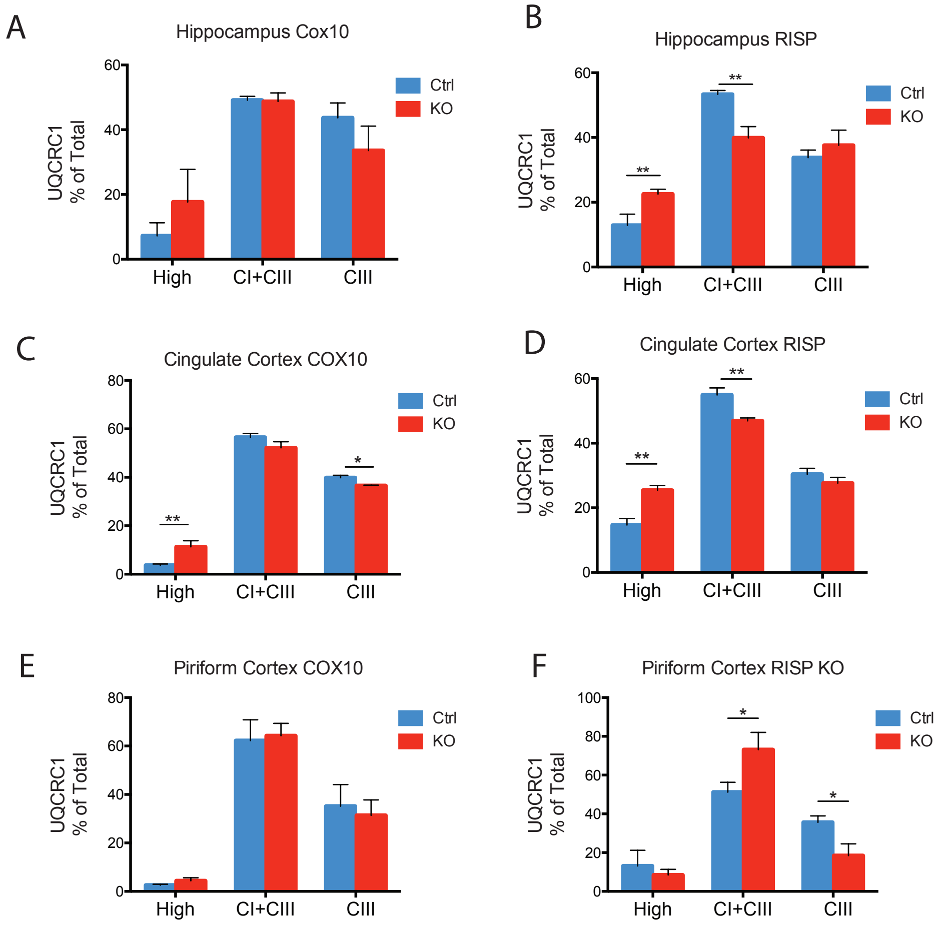

Fig S1. Quantification of UQCRC1 signal in blue native gels of mitochondria from hippocampus, cingulate cortex and piriform cortex of COX10 and RISP KO mice. Mitochondria were extracted for BN-PAGE from Ctrl and KO mice from: A) and B) Hippocampus form COX10 and RISP KO respectively; C) and D) Cingulate cortex from COX10 and RISP KO respectively; E) and F) Piriform cortex from COX10 and RISP KO. Signals obtained using the UQCRC1 antibody shown in Fig1 were quantified by densitometry using Image J and expressed as % of total signal. Bars represent mean and standard deviation. (*) p<0.05 and (**) p<0.01 indicate statistical significance, n=3.

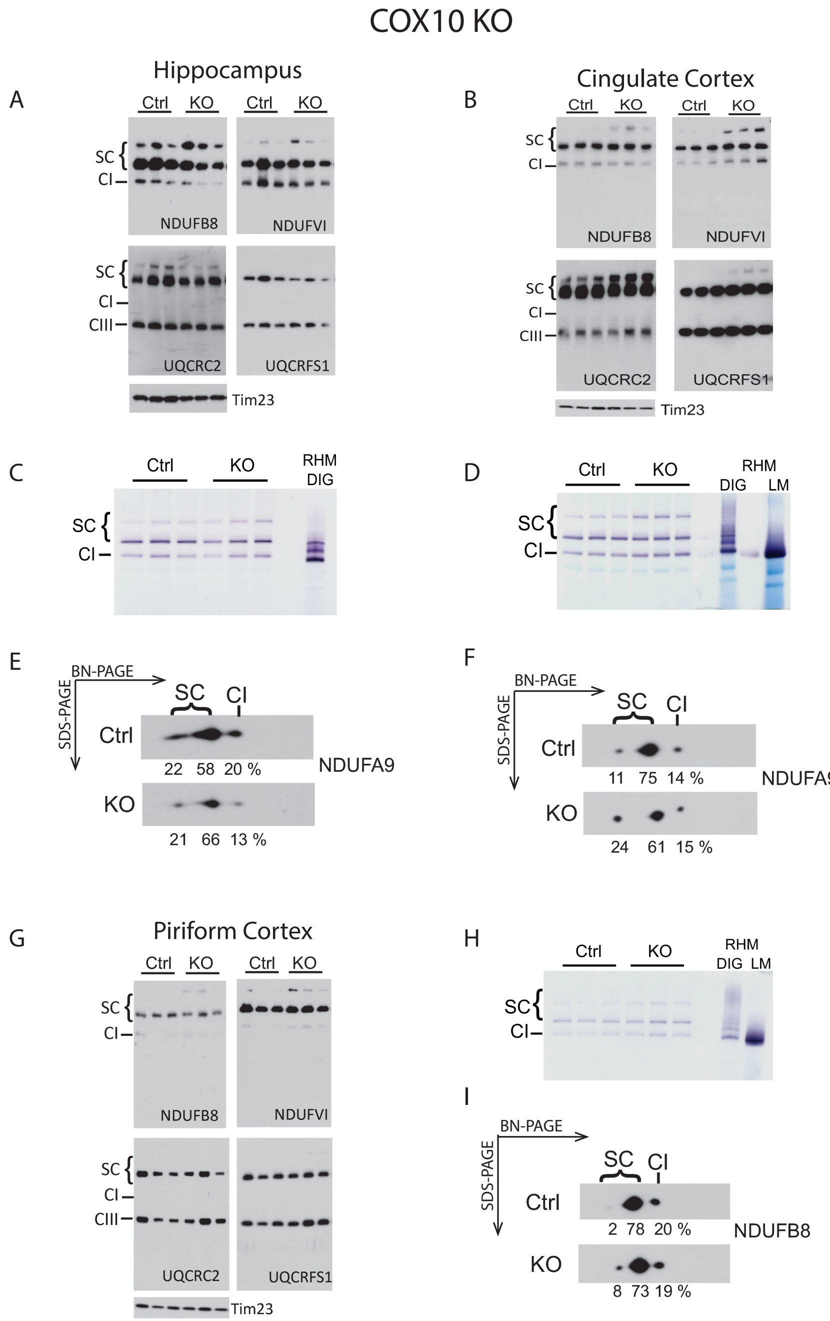

Fig S2. Analysis of mitochondria from hippocampus, cingulate cortex and piriform cortex from COX10 KO mice. A) B) G) BN-PAGE of mitochondria from control and COX10 KO mice from hippocampus, cingulate cortex and piriform cortex respectively using antibodies against CI (NDUFA9, NDUFB8 and NDUFV1) and CIII (UQCRC1, UQCRC2 and UQCRFS1/RISP) subunits. C) D) H) Complex I *in gel activity*. E) F) I) two dimension-BN-PAGE showing % of total signal for each spot.

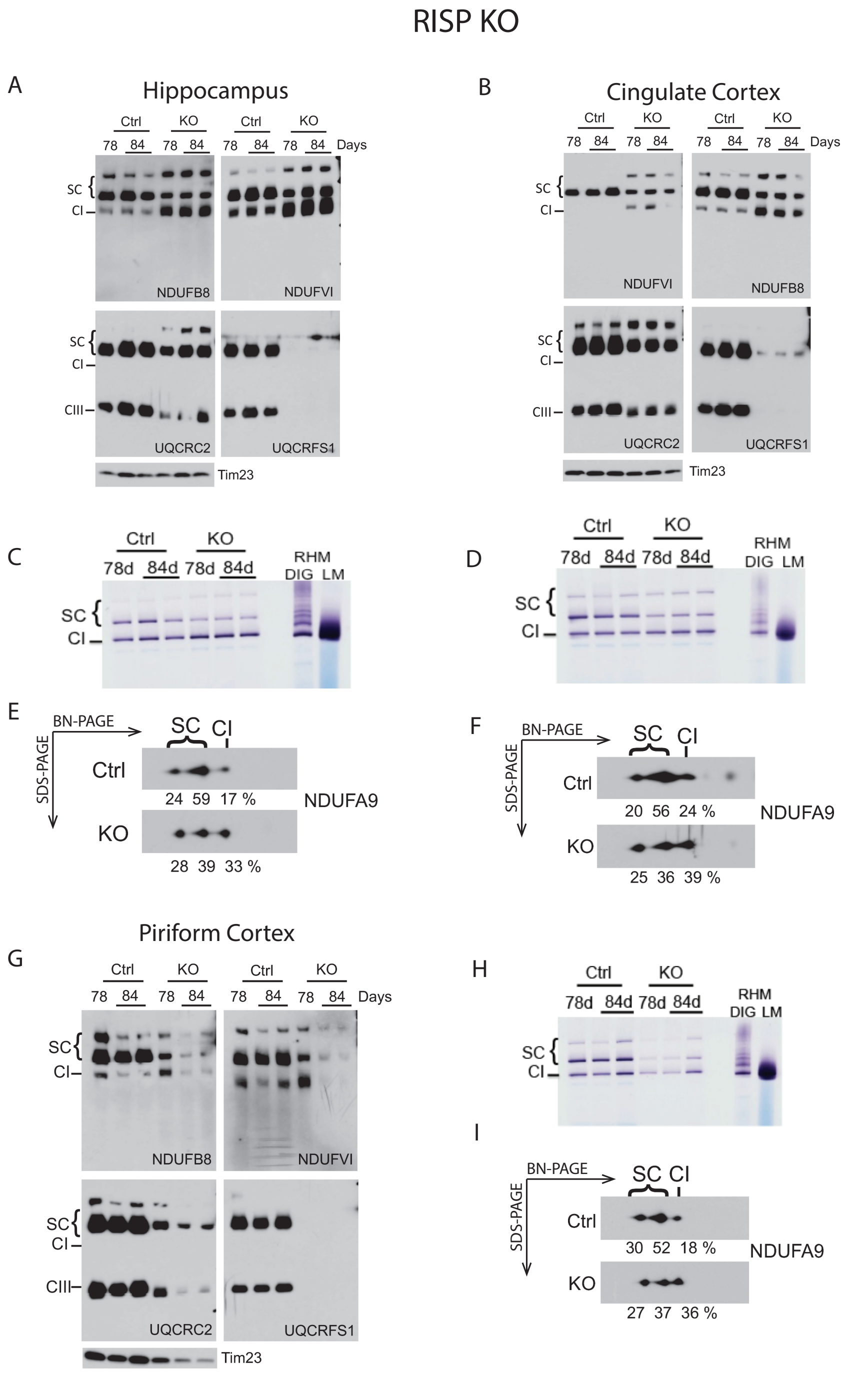

Fig S3. Analysis of mitochondria from hippocampus, cingulate cortex and piriform cortex from RISP KO mice. A) B) G) BN-PAGE of mitochondria from control and RISP KO mice from hippocampus, cingulate cortex and piriform cortex respectively using antibodies against CI (NDUFA9, NDUFB8 and NDUFV1) and CIII (UQCRC1, UQCRC2 and UQCRFS1 or RISP) subunits. C) D) H) Complex I *in gel activity*. E) F) I) two dimension-BN-PAGE showing % of total signal for each spot.

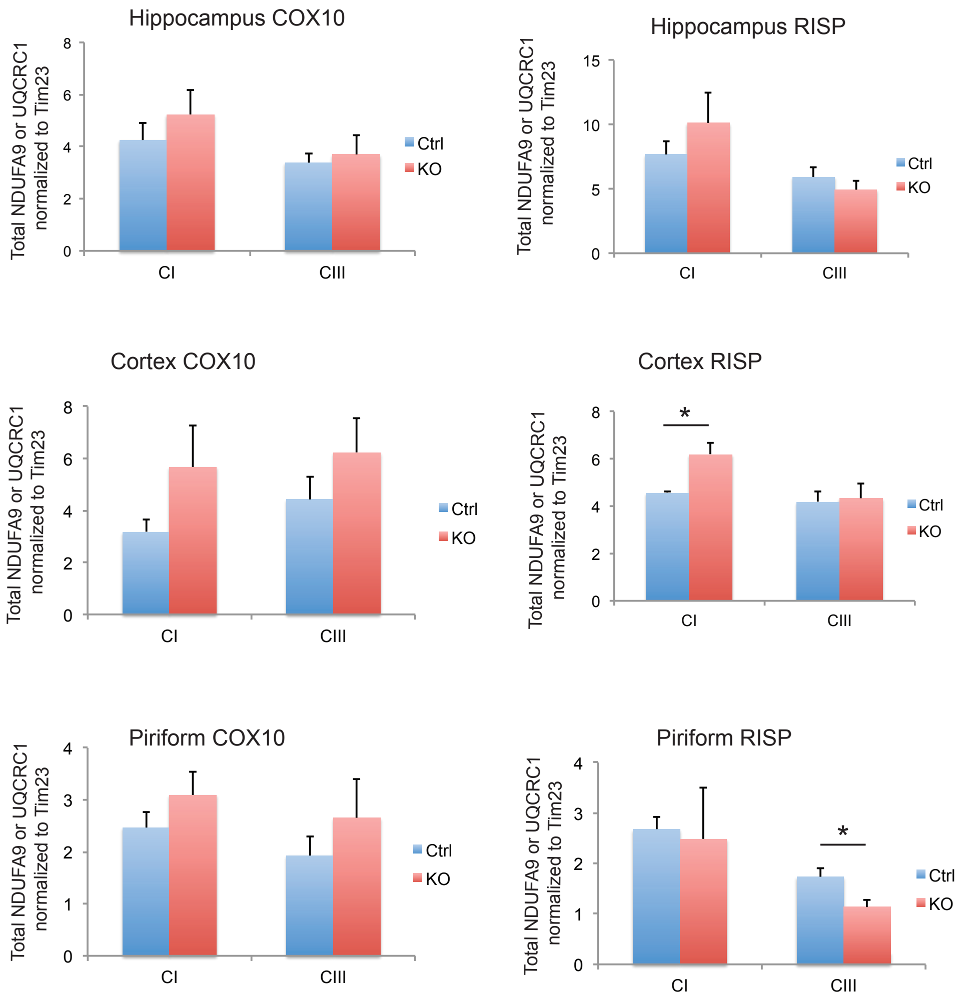

Fig S4. Quantification of total NADUFA9 and UQCRC1 signal in hippocampus, cortex and piriform cortex of COX10 and RISP KO. Total signals of NDUFA9 (CI) and UQCRC1 (CIII) blots from different brain regions in Fig 1 were quantified using Image J software and normalized to Tim23 signal. (*) $p<0.05$ indicates statistical significance, n=3.

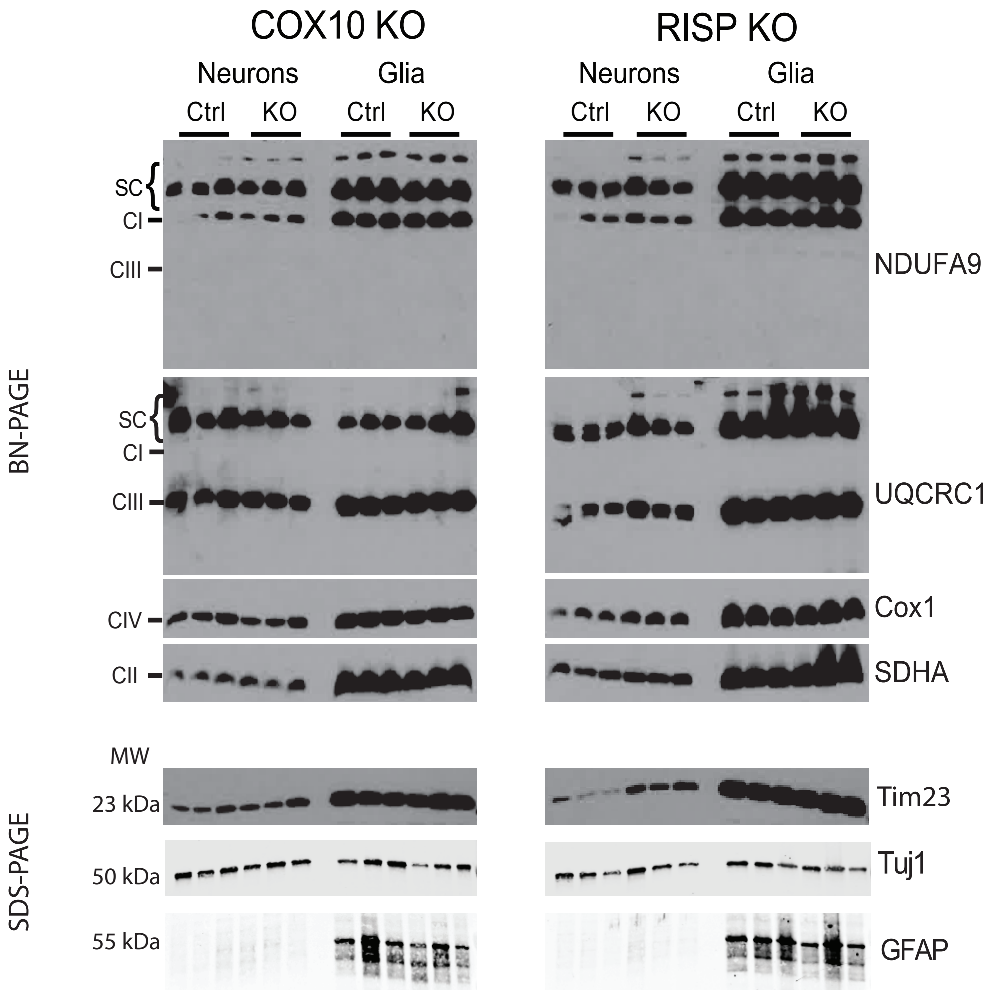

Fig S5. Mitochondrial supercomplexes in neuron and glial fraction isolated from COX10 and RISP KO. Neurons and glial cells were isolated from whole brains from control and KO mice using MACS technology and analyzed by BN-PAGE. Purity of cell fractions was assessed by blotting with antibodies against Tuj1 a neuronal marker and GFAP and astrocyte marker. Tim23 was used as loading control. Molecular weight (MW) of proteins are indicated.

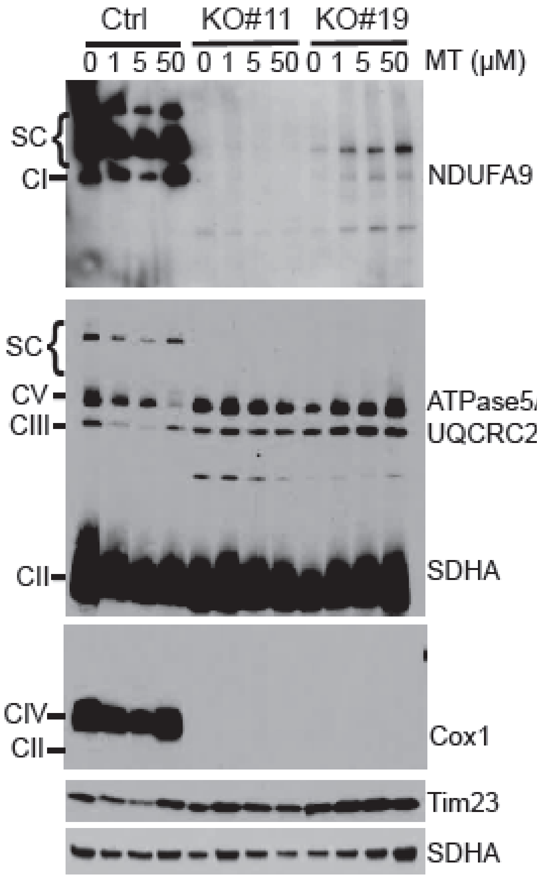

Fig S6. Mitochondrial supercomplexes in COX10 KO fibroblasts treated with mitoTEMPO. Control and COX10 KO mouse fibroblast (#11 and #19) were incubated with different concentrations of MitoTEMPO (MT) for 24hr. Cell homogenates were prepared for the SCs analyzed by BN-PAGE and western blot to detect SCs, CI, CIII, CIV and CII using antibodies against NDUFA9, ATPase5a, UQCRC1, Cox1 and SDHA subunits respectively. Tim23 was used as mitochondria loading control for BN-PAGE.
